# Influence of Different Housing Systems on Distribution, Function and Mitogen-Response of Leukocytes in Pregnant Sows

**DOI:** 10.3390/ani3041123

**Published:** 2013-12-03

**Authors:** Verena Grün, Sonja Schmucker, Christiane Schalk, Birgit Flauger, Ulrike Weiler, Volker Stefanski

**Affiliations:** Department of Behavioral Physiology of Farm Animals, Institute for Animal Husbandry and Animal Breeding, University of Hohenheim, Garbenstr. 17, 70599 Stuttgart, Germany; E-Mails: sonja.schmucker@uni-hohenheim.de (S.S); christiane_schalk@uni-hohenheim.de (C.S.); birgit.flauger@uni-hohenheim.de (B.F.); weiler@uni-hohenheim.de (U.W.); volker.stefanski@uni-hohenheim.de (V.S.)

**Keywords:** pig, group-housing, individual gestation crate, T cell subsets, lymphocyte proliferation, TNFα, IFNγ, cortisol

## Abstract

**Simple Summary:**

The European Union imposes housing of pregnant sows in social groups since 2013 for animal welfare reasons. Nevertheless, the consequences of different housing conditions for the immune system of pregnant sows remain poorly investigated. We therefore analyzed important aspects of blood cellular immunity and cortisol concentrations of sows either housed in individual crates or in a group during gestation. The results show that individually housed sows had lower T cell numbers, but higher cortisol concentrations. Obviously, common housing conditions can differentially affect key elements of the adaptive immune system and hormonal indicators of stress in pregnant sows.

**Abstract:**

In pig production, pregnant sows are either housed in individual crates or in groups, the latter being mandatory in the EU since 2013. The consequences of different housing conditions on the immune system are however poorly investigated, although immunological alterations may have severe consequences for the animal’s health, performance, and welfare. This study assessed measures of blood celluar immunity with special emphasis on T cells in pregnant German Landrace sows either housed in individual crates or in a social group. Blood samples were taken at four samplings *pre partum* to evaluate numbers of lymphocyte subpopulations, mitogen-induced lymphocyte proliferation and cytokine-producing T cells. Plasma cortisol concentrations were evaluated as an indicator of stress. We found lower blood lymphocyte numbers (*p* < 0.01) in individually housed as opposed to group-housed sows, an effect due to lower numbers of cytotoxic T cells, naive TH cells, and CD8^+^ γδ-T cells. Individually housed sows showed higher cortisol concentrations (*p* < 0.01), whereas lymphocyte functionality did not differ between sows of both housing systems. Possible implications and underlying mechanisms for the endocrine and immunological differences are discussed. We favor the hypothesis that differences in the stressfulness of the environment contributed to the effects, with crate-housing being a more stressful environment—at least under conditions of this study.

## 1. Introduction

In pig husbandry, pregnant sows are either housed individually (in gestation crates) or in social groups worldwide. In the European Union, group housing of pregnant sows is mandatory from four weeks after mating to one week before parturition since 2013 for animal welfare reasons (EU directive 2001/88/EC), since confinement in individual crates is presumed to be a more stressful condition [1]. The consequences of different housing systems on the immune system of sows are however poorly investigated—a surprising fact—considering that housing-associated alterations of the immune system may directly affect the animals’ health and welfare with possible economic disadvantages. 

It has been shown that housing sows in individual crates favors the development of stereotyped behavior [2,3], triggered by frustrating situations such as restricted environmental stimulation and limited opportunities to perform basic behaviors such as exploration and foraging [1,4]. The inability to resolve conflict with neighboring sows by active avoiding behavior or direct physical interaction was also presumed to cause frustration [4] and chronic stress [5,6]. Accordingly, cortisol concentrations were found to be higher in crate-housed than in group-housed sows, at least under certain conditions [1,6,7]. Moreover, a reduced muscle and bone strength and a higher susceptibility towards urinary tract infections was also reported in sows confined in individual crates during gestation [1,7]. On the other hand, housing of sows in groups requires mixing and regrouping of unfamiliar animals—especially in dynamic groups—that lead to agonistic interactions for hierarchy establishment [8,9,10]. As in many other mammalian species, social instability is a stressor that causes behavioral alterations [11,12,13], HPA-axis activation and increased glucocorticoid concentrations [9,12,14] in pigs. Studies in sows have shown that a social hierarchy is usually established within one week after forming a new group [8], and the majority of agonistic interactions occur within the first 48 hours [15]. Thus, a risk for prolonged chronic stress might be rather low for most members of a large group, although the time for complete social integration of a smaller subgroup into an existing group may take longer [16].

Many studies in rodents have convincingly demonstrated that stressful housing conditions can affect the immune system with substantial consequences for immune cell distribution and activity. Characteristic effects include a reduced number and function of blood T cells and an increased number of granulocytes [17,18,19]. Studies in pigs in this respect are relatively rare and have primarily focused on piglets or growing pigs. Damgaard *et al.* [20] reported higher neutrophil numbers in young pigs housed in groups with frequent exchange of group members as compared to groups with constant composition. Deguchi and Akuzawa [14] showed that mixing of unfamiliar piglets reduced mitogen-induced lymphocyte proliferation as compared to unmixed individuals. A similar effect was also observed by de Groot *et al.* [21] who found lower cell proliferation and lower IFNγ- and IL-10-production in antigen-stimulated lymphocytes from mixed barrows as compared to unmixed controls. 

Most studies that investigated housing conditions in sows focused on behavioral aspects, e.g., [3,13,22] and on consequences for fertility and reproduction [23,24], and only a few included immunological measures. Karlen *et al.* [2] and Broom *et al.* [4] reported higher lymphocyte numbers in pregnant sows when housed in large groups instead of individual crates. The effect on granulocytes however was inconsistent between the studies. Other studies failed to detect differences in leukocyte numbers [25,26], antibody titers following antigenic challenge [4,25], or acute phase cytokine response [27] between sows housed in individual crates or social groups. Taken together, current reports suggest that housing conditions have at least some effect on lymphocyte numbers in pregnant sows. However, the effects of housing on T cells including their functionally distinct subsets as well as on B and NK cells were not investigated so far, even though these subsets play an essential role for the immune defense of the organism [28,29]. It is important to recognize that the porcine immune system differs in several aspects from that of model species such as rats and mice. Unique characteristics exist in the T cell system with the occurrence of extrathymic CD8^+^ TH cells and a high proportion of blood γδ-T cells [29,30,31]. Extrathymic CD8^+^ TH cells are considered antigen-experienced and activated memory TH cells that origin from naive CD8^−^ TH cells which show an up-regulated response to foreign antigens by proliferation and the expression of CD8 molecules [29,31]. To note, porcine CD8^−^ γδ-T cells also seem to acquire CD8 during activation and maturation [29]. So far, γδ-T cells have been found in all vertebrates examined, but the abundance and tissue distribution of these cells vary within different species [32,33]. In mice and rats peripheral γδ-T cells represent 0.5%–2% of lymphocytes [32], whereas in 12-month old pigs they mount up to 23% among peripheral blood lymphocytes [31]. Although their role in immune defense is not yet clear [34], it becomes increasingly evident that these cells are important in the immune response against pathogens as they are able to perform different effector functions like e.g., cytotoxic activity and cytokine production [31,33].

The aim of this study was to compare the effect of two distinct housing systems for pregnant sows (confinement in individual crates and group-housing) on several measures of blood cellular immunity. In an experimentally controlled setting, by including, but not exactly mimicking elements that are common housing practice, we especially aimed to identify which elements of the relatively unique blood T cell subsets are sensitive to differences in housing. Proliferation assays were included to examine lymphocyte reactivity and cortisol measurements as an indicator of the stressfulness of the housing environment.

## 2. Experimental Section

### 2.1. Experimental Animals and Housing Conditions

All procedures were conducted according to the ethical and animal care guidelines and approved by the local authority for animal care and use. German Landrace sows were kept under controlled environmental conditions at the research swine center of the University of Hohenheim (Unterer Lindenhof, Eningen, Germany). For the experiments, a total of 33 multiparous sows (second till seventh pregnancy) were investigated in 11 independent replicates with three females each. Over a period of eleven weeks, three experimental sows per week were naturally mated with Piétrain boars and housed in identical individual cages (2.65 m × 1.95 m) until week 4 of gestation, when ultrasonography was performed to ensure pregnancy. Subsequently, one sow was randomly selected and further confined in a conventional individual gestation crate (CR; 2.25 m × 0.65 m), while the two other sows were introduced into a social group (GP). Group-housed sows were kept in an 83 m² area which was structured by five separated lying areas with solid floor accounting for approximately 60% of the total area. Straw was provided in both housing systems daily after removal of the soiled bedding material. Sows were fed twice a day with a standard diet for pregnant sows (135 g crude protein, 30 g crude fat, 57 g crude fiber, 54 g crude ash, 12.1 MJ per kg food) and had free access to water. Until day 84 of pregnancy, each sow obtained 2.5 kg food daily, thereafter 3.2 kg. In the GP, each sow had been fitted with an ear transponder system which allowed identification of the individual and computer controlled food delivery to a single-space electronic sow feeder (Compident VII; Schauer Agrotronic GmbH). All sows were kept in the same building to assure comparable environmental conditions (light exposure, ambient temperature, animal keeper, pathogen exposure). 

### 2.2. Experimental Procedure

The social group consisted of 22 pregnant sows (German Landrace) in which weekly two resident sows were replaced by two experimental animals (dynamic group). Before the start of the experimental phase, two trials with non-experimental sows were conducted to assure comparable conditions for all experimental animals. Individually housed sows were kept next to each other in closely adjacent crates separated by horizontal bars (16–20 cm distance) that mostly restricted physical contact. Sows chosen for CR-housing were directly introduced into their new housing system (Mondays, 0900 h), whereas sows for the GP were trained to use the electronic sow feeder before being introduced into their new housing system (Mondays, 1400 h). Prior to introducing new animals to the social group, straw was littered down for diversion. Sows were kept in each housing system for a period of eleven weeks and were transferred to farrowing crates thereafter. To maintain the group size of 22 animals, sows of the social group were replaced by non-experimental sows.

### 2.3. Blood Sampling and Treatment of Samples

Blood samples were collected from all animals 7, 6, 4, and 2 weeks *pre partum* on Tuesdays between 0900–1000 h by jugular vein puncture. Sows were restrained for blood sampling using a nose snare. Blood was collected in heparinized monovettes (Sarstedt, Nürnbrecht, Germany) and used for immunological measurements within 4 h after sampling. For cortisol measurements, plasma samples were stored at −20 °C until assayed. 

### 2.4. Body Mass and Medical Treatments

Each sow was weighed just before introduction into the new housing system and one week before parturition. All animals were regularly checked for the occurrence of lesions and injuries or an impaired health status. Claw and leg injuries as well as spontaneous diseases primarily occurred during the first 3 weeks after introduction. Four experimental sows of the social group were excluded from the experiment following leg injuries. These animals were replaced by non-experimental sows at the next regular integration cycle to maintain the standard group size of 22 sows. One individually housed sow had to be excluded from the study due to spontaneous abortion. The results in this study formed part of a larger research project regarding the effects of different gestation sow housing systems on physiological parameters of the offspring. Within this broader scope all sows were immunized with an experimental antigen seven and five weeks before parturition (results will be published elsewhere). Some of the sows received medical treatment before or during the sampling period, which did not influence the experimental outcome of the study (see Section 2.8). 

### 2.5. Flow Cytometry

Total white blood cell (WBC) counts were determined on a Z2 Coulter Counter (Beckman Coulter, Krefeld, Germany). The percentage of leukocyte and lymphocyte subpopulations was quantified by flow cytometry in a whole-blood three-color immunofluorescent antibody staining procedure referring to previous studies [35]. In detail, blood aliquots were incubated for 15 min at room temperature (RT) with different combinations of fluorochrome-labeled antibodies directed against the cell surface markers CD3 (clone PPT3), CD4 (clone 74-12-4), CD8α (clone 76-2-11), and CD172α (clone 74-22-15) (Biozol, Eching, Germany). Lysis of erythrocytes was performed through incubation (10 min, RT) with FACS lysing solution (BD Biosciences, Heidelberg, Germany). For analysis, labeled cells were washed and then resuspended in phosphate-buffered saline (PBS) supplemented by 2% FCS (Biochrom, Berlin, Germany) and 1% NaN_3_. 20,000 cells from each sample were analyzed at a FACSCalibur^TM^ flow cytometer using the software CellQuest Pro^TM^ (BD Biosciences, Heidelberg, Germany). Granulocytes were identified by their forward and side scatter characteristics. Monocytes, lymphocytes, and lymphocyte subpopulations were identified by their forward scatter characteristics and the combination of specific surface markers: CD3^+^ (T cells), CD3^+^CD4^+^CD8α^−^ (CD8^−^ T Helper cells; naive TH), CD3^+^CD4^+^CD8α^+^ (CD8^+^ T Helper cells; CD8^+^ TH), CD3^+^CD4^−^CD8α^high^ (cytotoxic T cells; CTL), CD3^+^CD4^−^CD8α^−^ (CD8^−^γδ-T cells), CD3^+^CD4^−^CD8α^low^ (CD8^+^γδ-T cells), CD3^−^CD8α^−^CD172^high^ (monocytes), CD3^−^CD8α^−^CD172^low^ (dendritic cells; DC), CD3^−^CD8α^−^CD172^−^ (B cells) and CD3^−^CD8α^+^CD172^−^ (natural killer cells; NK). Ratios of granulocytes:lymphocytes, CTL:TH cells, and γδ:αβ T cells were evaluated using absolute numbers of respective cell populations. Absolute numbers of cells were determined by WBC counts and percentages of immune cell subpopulations. 

### 2.6. Separation of Peripheral Blood Mononuclear Cells (PBMCs)

PBMCs were isolated using Leucosep^TM^ (Greiner Bio-One; Frickenhausen, Germany) according to the manufacturer’s protocol. In brief, Leucosep^TM^ tubes were filled with 15 mL of Biocoll separating solution. After centrifugation (30 sec, 1,000 × g; RT), 20 mL of heparinized blood were poured into the Leucosep^TM^ tube and diluted with 10 ml of PBS without Ca^2+^/Mg^2+^. Samples were centrifugated (15 min, 800 × g). The PBMC layer was transferred into a new centrifugation tube and washed once in RPMI 1640 medium by centrifugation (15 min, 400 × g). PBMC were resuspended in RPMI-10 (RPMI 1640 supplemented with 10% FCS and 50 µg/mL gentamycin) and cell concentration was determined by a Z2 Coulter Counter (Beckman Coulter, Krefeld, Germany) [17,18]. 

#### 2.6.1. Lymphocyte Proliferation

Activity of immune cells was examined *in vitro* using a mitogen-induced lymphocyte proliferation assay according to Stefanski *et al.* [18] with few modifications. T cell-specific mitogen concanavalin A (ConA, M 5050, Biochrom, Berlin, Germany) and T cell-dependent B cell mitogen, pokeweed mitogen (PWM, Sigma Aldrich, Munich, Germany) were used for stimulation of proliferation. The cell concentration was adjusted to 1.5 × 10^6^ cells per mL in RPMI-10. 100 µL of isolated PBMC (1.5 × 10^5^ cells per well) were pipetted into U-bottom 96-well cell culture plates (Neolab, Heidelberg, Germany) in triplicates per treatment and stimulated with 5 µg/mL of the appropriate mitogen or left without stimulation. After 44 h of incubation (37 °C, 5% CO_2_), cells were pulsed with 0.5 µCi tritiated thymidine ([6-3H], PerkinElmer, Rodgau, Germany) per well. Following another 24 h of incubation, cells were harvested on glass fiber filters (Skatron, Lier, Norway) and radioactivity was measured by a liquid scintillation analyzer (PerkinElmer, Rodgau, Germany). The mean of counts per minute (cpm) was calculated for each triplicate and the ∆ cpm for ConA and PWM was determined (∆ cpm = stimulated cells − unstimulated cells) for each individual.

#### 2.6.2. Intracellular Cytokine Staining

Intracellular staining was performed referring to Zelnickova *et al.* [36] and Mascher *et al.* [37] with the following modifications: PBMC were resuspended in RPMI 5 (RPMI 1640 supplemented with 5% FCS and 50 µg/mL gentamycin). 10^6^ PBMC per well were cultured in 96-well flat-bottom culture plates (Orange scientific, Braine l’Alleud, Belgium) and either remained unstimulated or were stimulated with 40 ng/mL phorbol 12-myristate 13-acetate (PMA) and 2 µg/mL ionomycin for 4 h (5% CO_2_, 37 °C). Brefeldin A was added to unstimulated and stimulated PBMC at a concentration of 1 µg/mL at the same time to inhibit cytokine secretion. After incubation, cells were harvested and fixated for 20 min (RT) in 0.5% formaldehyde buffer (PBS, 2mM EDTA, 0.5% FCS, 0.5% Roti-Histofix formaldehyde; Karl Roth GmbH, Karlsruhe, Germany). Subsequently, cells were permeabilizied (15 min, RT) using saponin buffer (PBS, 2 mM EDTA, 0.5% FCS, 0.05% saponin) and stained intracellular with antibodies directed against the cytokines IFNγ (clone P2G10) and TNFα (clone MAb11) (both, BD Biosciences, NJ, USA) as well as antibodies directed against the cell surface marker CD3 (clone PPT3, Acris Antibodies, Herford, Germany). Following intracellular staining, cells were resuspended in PBS (supplemented by 2% FCS and 1% NaN_3_) and analyzed at a FACSCanto^TM^ flow cytometer (BD Biosciences, NJ, USA) using the software BD FacsDiva^TM^ and FlowJo 7.6.5 (Tree Star, OR, USA) by evaluating percentages of cytokine-positive T cells. To determine PMA/ionomycin-induced cytokine production, the background of cytokine-producing T cells in the unstimulated controls were subtracted from the frequencies of cytokine-producing T cells after PMA/ionomycin stimulation (∆% = % cytokine^+^ T cells within stimulated cells − % cytokine^+^ T cells within unstimulated cells). Unless otherwise stated, all reagents mentioned in 2.6 were obtained from Biochrom (Berlin, Germany). EDTA, PMA, ionomycin, brefeldin A and saponin were obtained by Sigma Aldrich (Munich, Germany). 

### 2.7. Cortisol Determinations

Plasma cortisol concentrations were determined radioimmunologically as described by Claus and Weiler [38] with the following modifications. Before radioimmunological determination, an extraction of samples (5 µL diluted with 100 µL aqua bidest) with ethyl acetate (Applichem, Darmstadt, Germany) was carried out. The antiserum for cortisol determination had been raised against HCO-21-HS-BSA, and revealed a cross-reactivity of 6.8% with desoxycorticosterone, 12.1% with corticosterone, 1.2% with aldosterone, and 1.4% with progesterone. It was used at a final dilution of 1:71000 in the assay. The mean recovery rate of [3H]-cortisol after extraction was 91.2 ± 2.4%. The recovery rate of cortisol in spiked samples ranged from 111.9 to 99.1% for samples spiked with 10, 25 or 50 ng cortisol per mL plasma. The coefficients of the interassay variation ranged between 14.1 and 8.2% depending on endogenous concentrations (15.1 ng/mL and 37.4 ng/mL, respectively) and 2.4% for the intraassay variation.

### 2.8. Statistical Analysis

Statistical analyses were performed on data of 18 group-housed sows and 10 individually kept sows using IBM SPSS Statistics 20 (IBM; Eningen, Germany) unless stated otherwise. Both groups did not differ in age, body mass, and number of litters per sow (unpaired student’s *t*-test). The effects of the housing conditions on immunological and endocrine parameters were analyzed by a repeated measures analysis using linear mixed-effect models (type III) with a first-order autoregressive covariance structure for the factors housing and gestational stage and their interaction. Gestational stage was defined as 7, 6, 4, and 2 weeks *pre partum* to analyze alterations during progressing pregnancy. For each model, normal distribution and variance homogeneity were evaluated with a Shapiro-Wilk test and a Levene’s test of the residuals. To achieve approximately normal distribution, logarithmic transformation was used for those parameters that were not normally distributed or where variance homogeneity could not be assumed. Each model was corrected for sampling duration, season and age by including those parameters as covariates. The covariates were excluded from the model if no significant effects (*p* < 0.05) could be detected. Weight and number of litters were not included into the model, because they were both positively and significantly correlated with age. If linear mixed-effect models revealed a significant effect of housing, the model was calculated for each week separately to determine at which stage of gestation this effect occurred. Significant differences among gestational stage were tested *post hoc* using the Bonferroni multiple testing procedure. To evaluate the potential influence of medicamentous treatments of diseases on investigated parameters, linear mixed-effect models were first performed including data of respective animals, and then in a second model excluding these data. As we could not detect any effect of diseases and medicamentous treatments on any parameter tested, data of respective individuals were included into the final models. For all comparisons, *p* < 0.05 was considered as significant and 0.05 < *p* < 0.1 as a tendency. Data are presented as mean ± SEM without transformation. 

**Figure 1:**
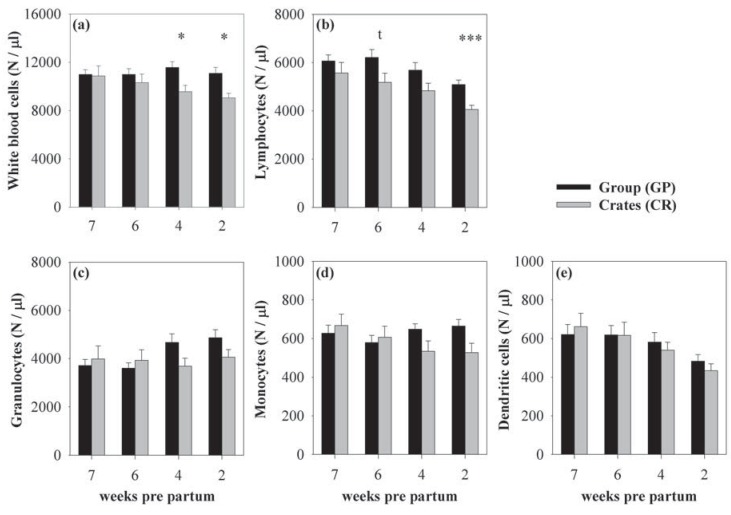
Mean (±SEM) numbers of white blood cell (WBC) and leukocyte subpopulations in group-housed sows (GP, black bars) and sows kept in individual crates (CR, grey bars) 7, 6, 4, and 2 weeks *pre partum*. The housing system significantly affected numbers of WBC (**a**) 4 and 2 weeks *pre partum* and numbers of lymphocytes (**b**) 2 weeks *pre partum*, with both being higher in GP-housed sows. Tendentially higher lymphocyte numbers were also found 6 weeks *pre partum* in GP-housed sows. No effect could be found for the number of granulocytes (**c**), monocytes (**d**), and dendritic cells (**e**). Tendencies are stated as ^t^*p* < 0.1; Asterisks indicate significant differences between the housing systems at the respective stage of gestation: *p < 0.05; ***p < 0.001; (CR: n = 10; GP: n = 18 except at 7 weeks *pre partum*: GP: n = 17).

## 3. Results

### 3.1. Effects of Housing and Gestational Stage on Number and Ratio of Blood Immune Cells

**Figure 2:**
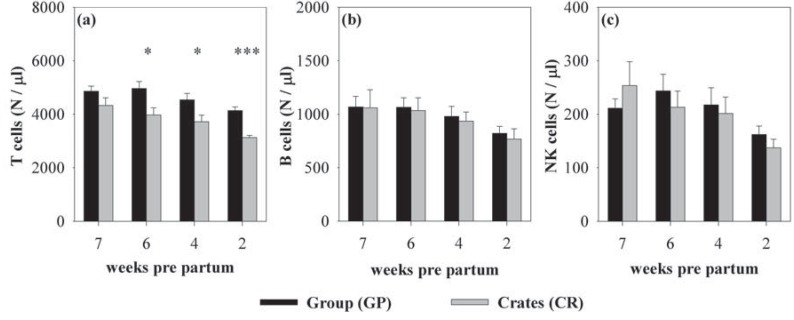
Mean (±SEM) numbers of lymphocyte subpopulations in group-housed sows (GP, black bars) and sows kept in individual crates (CR, grey bars) 7, 6, 4, and 2 weeks *pre partum*. Numbers of T cells (**a**) were significantly higher in GP-housed sows 6, 4, and 2 weeks *pre partum*, whereas numbers of B cells (**b**) and NK cells (**c**) were not influenced by the housing system. Asterisks indicate significant differences between the housing systems at the respective stage of gestation: *p < 0.05; ***p < 0.001; (CR: n = 10; GP: n = 18 except at 7 weeks *pre partum*: GP: n = 17).

**Figure 3:**
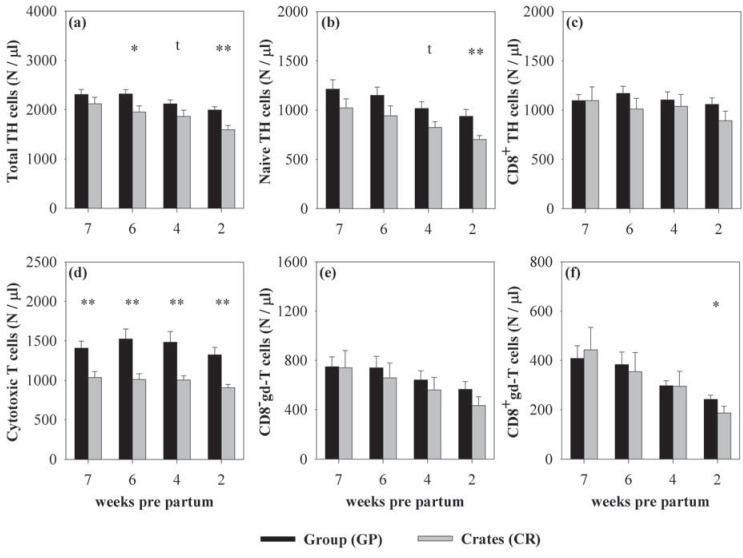
Mean (±SEM) numbers of T cell subpopulations in group-housed sows (GP, black bars) and sows kept in individual crates (CR, grey bars) 7, 6, 4, and 2 weeks *pre partum*. Total TH cells (**a**) were significantly affected by the housing system 6, and 2 weeks *pre partum*. This effect was mainly due to higher numbers of naive TH cells (**b**) in GP-housed sows 2 weeks *pre partum*, whereas the number of CD8^+^ TH cells (**c**) was not influenced by the housing system. Cytotoxic T cells (**d**) were significantly higher at all stages of gestation in GP-housed sows compared to CR-housed sows. In γδ-T cells CD8^−^ γδ-T cells (**e**) were not influenced, whereas CD8^+^ γδ-T cells (**f**) were significantly higher in GP-housed sows 2 weeks *pre partum*. Tendential differences were also detected 4 weeks *pre partum* for the number of total TH cells and naive TH cells, both of them being higher in GP-housed sows. Tendencies are stated as ^t^*p* < 0.1; Asterisks indicate significant differences between the housing systems at the respective stage of gestation: *p < 0.05; **p < 0.01; (CR: n = 10; GP: n = 18 except at 7 weeks *pre partum*: GP: n = 17).

In the following text, the main effects of housing and gestational stage on leukocyte and lymphocyte subsets are shown. Statistical details for housing comparisons at certain gestational stages are reported in the respective figure legends. Group-housed sows showed higher numbers of WBC (F_(1,29.14)_ = 4.71, *p* < 0.05) than individually housed sows (Figure 1a). These differences were due to higher lymphocyte numbers (F_(1,33.54)_ = 8.51, *p* < 0.01, Figure 1b) as numbers of granulocytes, monocytes, and DC did not differ between the housing systems (Figure 1c–e). Subsequent analyses of lymphocyte subsets revealed that the effect in lymphocytes was caused by higher numbers of T cells (F_(1,34.17)_ = 12.34, *p* < 0.01) in group-housed sows, whereas B cell and NK cell numbers did not differ between the housing systems (Figure 2a–c). Detailed analyses of T cell subsets indicated that TH cells (F_(1,31.68)_ = 9.91, *p* < 0.01, Figure 3a), CTL (F_(1,31.88)_ = 17.14; *p* < 0.001, Figure 3d), and CD8^+^ γδ-T cells (F_(1,30.05)_ = 2.92, *p* = 0.098, Figure 3f) were higher in sows housed in the social group, whereas CD8^−^γδ-T cells did not differ between the housing systems (Figure 3e). The effect on TH cells was mainly based on differences in naive TH cells (F_(1,27.71)_ = 3.86, *p* = 0.06, Figure 3b), whereas CD8^+^ TH cells were not affected (Figure 3c). The different effect on T cell subsets in both housing systems resulted in an altered TH:CTL ratio, which was higher in individually housed sows (F_(1,27.44)_ = 4.25; *p* < 0.05). The effect, however, did not reach level of significance when calculated for each week separately (*p* < 0.1 at 7, 6 and 4 weeks *pre partum*). The ratios of granulocytes:lymphocytes and γδ:αβ T cells were not influenced by the housing system [39].

To analyze alterations during progressing pregnancy, the effects of gestational stage on immune cell subsets were evaluated. Indeed we found a decline of lymphocyte (F_(3,76.73)_ = 7.42, *p* < 0.001) and DC numbers (F_(3,77.43)_ = 11.02, *p* < 0.001) with progressing pregnancy. Decreasing lymphocyte numbers were due to T cells (F_(3,77.54)_ = 8.37, *p* < 0.001), B cells (F_(3,73.41)_ = 4.24, *p* < 0.01), and NK cells (F_(3,75.91)_ = 4.44, *p* < 0.01). Detailed analysis of T cell subsets indicated that TH cells (F_(3,73.25)_ = 6.97, *p* < 0.001), γδ-T cells (F_(3,75.76)_ = 9.93, *p* < 0.001) as well as the TH:CTL ratio (F_(3,75.65)_ = 2.32, *p* < 0.082) declined with progressing pregnancy. The effect in TH and γδ-T cells was due to decreasing numbers of naive TH cells (F_(3;74.89)_ = 7.31, *p* < 0.001), and CD8^−^ γδ-T cells (F_(3,75.34)_ = 5.87, *p* < 0.01). Other immune cell subsets such as granulocytes, monocytes, CTL, CD8^+^ TH cells, CD8^+^ γδ-T cells as well as the ratios of granulocytes:lymphocytes and γδ:αβ T cells were not affected by gestational stage. Furthermore, there were no housing × gestational stage interactions, except a trend in monocytes (F_(3,71.17)_ = 2.32, *p* < 0.082) with increasing numbers in GP-housed and decreasing numbers in CR-housed sows as gestation progressed. Therefore the direction and magnitude of change over time was comparable in both housing systems.

### 3.2. Effects of Housing and Gestational Stage on Lymphocyte Functionality

To evaluate the reactivity of antigen-experienced lymphocytes in group- and individually housed sows, ConA- and PWM-induced lymphocyte proliferation as well as the frequency of IFNγ- and TNFα-producing T cells upon PMA/ionomycin stimulation was analyzed. Lymphocyte proliferation was not affected by housing, gestational stage or housing × gestational stage interaction (Table 1). Moreover, neither the frequencies of IFNγ- and TNFα-producing T cells nor the frequency of IFNγ/TNFα double-producing T cells upon PMA/ionomycin stimulation was influenced by the housing system or the housing × gestational stage interaction. Gestational stage, however, tendentially affected the frequency of IFNγ-producing T cells (F_(3,47.51)_ = 2.54, *p* = 0.068) and significantly affected the frequency of IFNγ/TNFα double-producing T cells (F_(3,47.09)_ = 2.91, *p* < 0.05), but these effects were not apparent after Bonferroni correction (Table 1).

**Table 1:** Influence of housing, gestational stage and housing × gestational stage interaction on lymphocyte proliferation (∆ cpm) after ConA and PWM stimulation and on intracellular cytokine production (IFNγ, TNFα,) among T cells after PMA/ionomycin stimulation 7, 6, 4, and 2 weeks *pre partum*. Values are expressed as mean ± SEM.

Parameter		Weeks pre partum				*p* values		
		7	6	4	2	Housing	Gestational stage	Housing × gestational stage
**Lymphocyte proliferation**								
∆ cpm ConA	GP	50294 ± 2998	48318 ± 3584	38945 ± 2289	39431 ± 3486	n.s.	n.s.	n.s.
	CR	48847 ± 3763	47777 ± 3926	41703 ± 3941	40191 ± 4459			
∆ cpm PWM	GP	67368 ± 2530	69127 ± 2737	73542 ± 4213	70502 ± 2959	n.s.	n.s.	n.s.
	CR	74993 ± 3982	73998 ± 4505	71465 ± 4086	71596 ± 3826			
**∆% of cytokine-producing T cells among all T cells**								
IFNγ^+^	GP	11.4 ± 2.6	8.6 ± 1.6	11.3 ± 1.7	12.2 ± 1.9	n.s.	<0.1	n.s.
	CR	12.1 ± 3.0	10.5 ± 2.5	14.1 ± 3.0	12.8 ± 3.5			
TNFα^+^	GP	28.7 ± 5.8	27.0 ± 3.7	30.5 ± 2.7	32.8 ± 4.0	n.s.	n.s.	n.s.
	CR	31.3 ± 7.6	29.2 ± 5.0	37.2 ± 7.4	28.1 ± 6.8			
IFNγ^+^/TNFα^+^	GP	10.1 ± 2.4	7.9 ± 1.4	10.0 ± 1.5	10.8 ± 1.8	n.s.	<0.05	n.s.
	CR	10.3 ± 3.1	8.9 ± 2.3	12.2 ± 2.6	11.6 ± 3.4			

GP: n = 18; CR: n = 10.

### 3.3. Effects of Housing and Gestational Stage on Plasma Cortisol Levels and Body Mass

**Figure 4:**
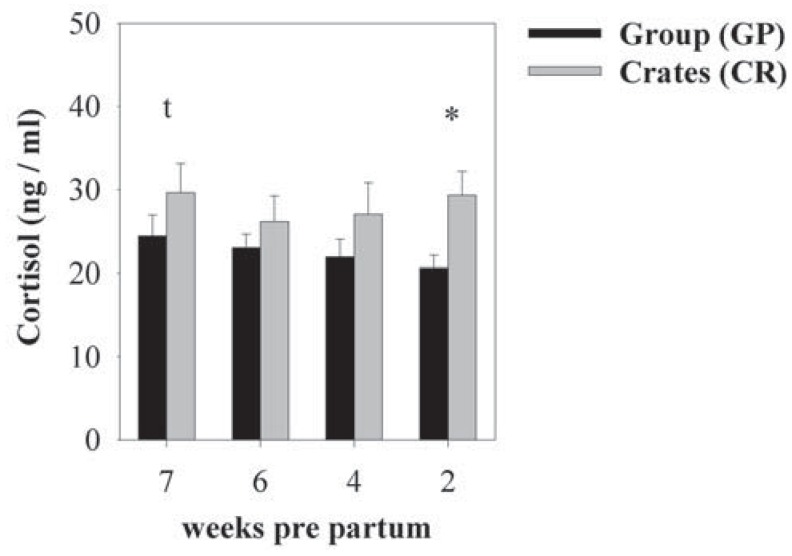
Mean (±SEM) plasma cortisol concentrations (ng/mL) in group-housed sows (GP, black bars) and sows kept in individual crates (CR, grey bars) 7, 6, 4, and 2 weeks *pre partum*. Sows kept in CR showed significantly higher plasma cortisol concentrations 2 weeks *pre partum*. A tendential effect was also detected 7 weeks *pre partum* with cortisol concentrations being higher in CR-housed sows. Tendencies are stated as ^t^*p* < 0.1; Asterisks indicate significant differences between the housing systems at the respective stage of gestation: *p < 0.05; (GP: n = 18; CR: n = 10).

Individually housed sows showed higher plasma cortisol concentrations during the whole sampling period than group-housed sows (F_(1,35.89)_ = 7,82, *p* < 0.01, Figure 4). Gestational stage and housing × gestational stage interaction had no effects on plasma cortisol levels [39]. Furthermore, body mass (GP: 258.8 ± 5.8 kg; CR: 252.3 ± 5.9 kg) and body mass gain (GP: 49.6 ± 2.2 kg; CR: 48.9 ± 3.7 kg) did not differ significantly between the housing systems at the end of the experimental procedure. 

## 4. Discussion

Two different housing conditions for pregnant sows are used worldwide—either confinement in individual crates or housing in social groups. Although group-housing of sows has been mandatory in the EU since 2013, the consequences of different housing systems on the immune system are still poorly investigated. In the present study we compared the effect of individual crate-housing *vs.* group-housing on key aspects of blood cellular immunity in pregnant sows under experimentally controlled conditions. As the main result, we demonstrated that several T cell subsets were lower in individually housed than in group-housed sows, while their cortisol levels were higher. 

### 4.1. Housing-Associated Alterations of the Immune System

The low blood lymphocyte numbers in individually housed sows as compared to sows kept in large groups generally agree with previous studies [2,4], but lymphocyte subsets have not been analyzed in relation to housing systems before. Our results indicate that housing-related differences in lymphocyte numbers between individually and group-housed sows are mainly due to lower T cell numbers, especially of CTL, naive TH cell and CD8^+^ γδ-T cell subsets. Possible mechanisms which may account for lowered T cell numbers in the circulation include apoptosis, a reduced T cell release from the thymus and/or an altered distribution pattern of immune cells. The first two factors would certainly contribute to systemically reduced cell numbers of CTL and naive TH cells in individually housed sows, with potentially negative consequences for the immune response towards foreign antigens and the resistance to viral infections [31]. Apoptosis of immune cells has mainly been reported following severe stress [40,41,42,43], a condition which we consider unlikely for individually housed sows of this study, since typical stress indicators were not affected (body mass, granulocyte numbers, lymphocyte proliferation). Although we cannot completely rule out apoptosis as a contributing factor, we assume that the observed differences might reflect a different migration pattern of T cells. T cells of individually housed sows may have left the blood and accumulated in secondary lymphoid organs or the bone marrow as presumed in other species [18,42,44]. Moderately lower numbers of lymphocyte subsets in individually housed sows should therefore not be prematurely interpreted as a negative immunological outcome. On the contrary, the redistribution of cells from the blood to the organs may enhance immune function in target tissues [45] or even facilitate the meeting of T cells with antigen-presenting cells in secondary lymphoid organs [42], thus causing a favorable condition for the initiation of an immune response in individually housed sows. Obviously, future studies should investigate whether the differences in the immune status between individually and group-housed sows affect disease resistance or impair the success of vaccination. 

### 4.2. Housing-Associated Alterations of Plasma Cortisol

The concentrations of plasma cortisol in both housing systems corresponded well or were lower than the concentration ranges observed in previous reports on sow housing [6,26], suggesting that probably none of these two housing conditions represents a highly stressful environment for pregnant sows. One problem when comparing plasma cortisol concentrations across different studies, however, is due to the fact that absolute values are always influenced by a large number of intrinsic (genotype, age, stage of pregnancy, circadian rhythm) and environmental (temperature, light regime, blood sampling method) factors. Absolute cortisol concentrations can therefore hardly be compared between studies, especially if rather moderate reactions to environmental conditions are of interest. Relative differences in cortisol concentrations between experimental groups within a study nevertheless often represent a valuable indicator of stress [46]. In the present report, cortisol concentrations were on average about 25% higher in sows housed in individual crates than in group-housed sows, a magnitude that definitely exceeds the normal range of variation and therefore strongly suggests a certain stress level. As body mass development—an indicator for very stressful conditions—did not differ between individually and group-housed sows, the cortisol increase in individually housed sows probably reflects a rather mild stress effect. The different effect on cortisol corresponds well with previous results of Barnett *et al.* [6] who reported higher cortisol concentrations in sows kept in crates with horizontal bars compared to group-housed sows. It is well known that the housing of sows in individual crates limits the animals’ opportunity to display several behavioral patterns and impedes the establishment of a social hierarchy [1], both conditions that were presumed to cause frustration [4] and psychosocial stress [6].On the other hand, several studies reported similar cortisol concentrations in crate-housed and group-housed sows [2,5,25,26], which might indicate that cortisol concentrations in crate-housed sows are influenced by the stall design. Indeed, high cortisol concentrations under individual housing conditions particularly occur if sows are kept in crates with horizontal bars [6] as in the present study, whereas housing in crates with vertical bars resulted in similar [6] or lower [47] cortisol concentrations compared to social groups. We therefore suggest differences in the crate design as well as different group-housing conditions (space availability, group size) to be responsible for the contradictory outcome of this study compared to certain previous findings. For this reason, it should, however, not be concluded that the lower cortisol concentrations in group-housed sows of this study mean a non-stressful environment during group-housing in general. Indeed, social instability and grouping of unfamiliar animals as required in group housing systems usually represent stressful conditions often associated with an activation of the HPA-axis in pigs [9,12,14,21]. For such conclusions to be made a group presumably representing a less stressful situation should be tested in the future (e.g., a stable group of acquainted sows) and compared to the dynamic group studied here. 

### 4.3. Factors Contributing to the Immunological Effects

In the following we will discuss three possible factors which may have contributed to the immunological differences between individually and group-housed sows: stress, environmental differences, and physical activity. It is well known that stress and high cortisol concentrations reduce the number of T cells in the circulation [48,49,50]. The reduced number of T cells in individually housed sows principally agrees with the concept of a stress-induced immunomodulation. However, since stress-induced HPA-axis activation is often associated with the suppression of lymphocyte proliferation [14,18,21,51] and with TH1 cytokine production [49,52,53] under many conditions, it appears at first glance surprising that the lymphocyte functionality was not inhibited in individually housed sows. However, pregnancy is associated with profound immunological alterations [54,55] and was often presumed to reduce or abrogate several aspects of normal stress-induced immunomodulation [9,56] including the suppression of lymphocyte function and the release of granulocyte numbers [2,9,35].

An alternative interpretation is that group-housed sows live in a much richer environment for exposure to pathogens and possible injury and therefore display an immunological phenotype (more T cells) commensurate with a higher rate of pathogen defense. We cannot completely rule out this interpretation, although we consider it unlikely. All animals were kept in the same building in close proximity to ensure comparable environmental conditions. Moreover, a higher rate of immune defense against bacteria and other microorganisms should be most notably reflected in higher number of innate immune cells in the circulation (granulocytes, monocytes), which was not the case in the present study. 

Differences between sows of the two housing systems certainly also exist with respect to the amount of physical activity, with a higher activity level in group-housed sows compared to sows kept in individual crates. Nonetheless, physical activity can also be ruled out as major factor for two reasons: First, most immune cell alterations become evident only after strenuous or long-term exercise [57,58], a level which is certainly not reached by the group-housed sows at the experimental phase when blood samples were taken. Secondly, strenuous exercise usually decreases lymphocyte numbers and enhances glucocorticoid concentrations [57,58], which is opposite to what was observed in group-housed sows. Weighing the arguments outlined above, we consider differences in the stressfulness of the environment as the most likely factor determining the effects observed in individually *vs.* group-housed sows. However, it should also be considered, that in this study we compared the effect of two housing systems completely contrary to each other. Further studies should thus analyze if the observed effects also occur when manipulating the group conditions (group size, social stability or floor space allowance).

### 4.4. Immunological Alterations During Pregnancy

According to the general picture of pregnancy-induced immune alterations, several immune cell populations were affected by the stage of gestation. Normal pregnancy involves an activation of the innate and a down-regulation of the adaptive immune response [9,35,59,60,61]. The activation of the innate immune system is presumed to compensate the weakened specific immune response which is down-regulated to protect the fetus from harmful maternal immune activity [55,62]. The present data agree with this picture, since the number of T, B and NK cells declined towards the end of pregnancy. The effect in T cells was mainly due to decreasing numbers of naive TH and CD8^−^γδ-T cells which indicates a down-regulation especially of the naive T cell function during pregnancy. A new interesting finding is the lower frequency of IFNγ- and IFNγ/TNFα-producing T cells 6 weeks *pre partum.* Alterations of the maturing fetal immune system such as the differentiation of CD4^+^CD8^−^ and CD4^−^CD8^+^ thymocytes [31] or increasing placental estrogen or progesterone production [63,64] at this gestational stage might contribute to these effects. In this study we did not find an increase in granulocyte numbers, but it is easily possible that this increase had occurred earlier during gestation, *i.e.*, before the sampling period started. Similar to humans [60], however, we found decreasing numbers of NK cells during late pregnancy, which is believed to protect the fetus from NK cell activity-associated abortions and fetal damages [55]. 

## 5. Conclusions

One important conclusion from this study is that the housing environment indeed has long-term consequences for the immune and the endocrine system in pregnant sows. It is clear that two rather distinct housing conditions were tested and different factors may have contributed to the observed effects. Obviously, future studies must identify the key variables underlying the effect of housing on the immune system in pregnant sows. Based on the current findings we favor the hypothesis that the stressfulness of the environment is a crucial factor. One important direction currently underway is to manipulate the group housing conditions with respect to animal size or group stability. Future studies are also required to evaluate the significance of the immunological effect. Low T cell numbers in the circulation might reflect altered cell migration patterns that should not prematurely be equated with reduced immune-competence of sows kept in individual crates. Further studies are needed to assess whether the observed immunological differences impair the success of vaccinations or result in increased susceptibility towards diseases. 
